# Massive Localized Lymphedema Arising from Abdominal Wall: A Case Report and Review of the Literature

**DOI:** 10.1155/2015/375090

**Published:** 2015-08-31

**Authors:** Teodóra Tóth, Yi-Che Chang Chien, Sándor Kollár, Ilona Kovács

**Affiliations:** Department of Pathology, Kenézy Gyula County Hospital, Bartók Béla út 2-26, Debrecen 4031, Hungary

## Abstract

Massive localized lymphedema (MLL) is a rare pseudosarcomatous lesion due to localized lymphatic obstruction from variable causes. It is most common on medial aspect of thigh and inguinal region. Abdominal localization is rare and may cause clinical diagnostic confusion with other malignant tumors due to its large size. We report a case of abdominal wall MLL of a 56-year-old male patient under clinical suspicion of well differentiated liposarcoma. The literature search and differential diagnosis will be addressed. In doubt cases, immunohistochemical stain or fluorescent in situ hybridization can help to separate this entity from the other mimickers.

## 1. Introduction

Massive localized lymphedema (MLL) is a rare pseudosarcomatous lesion that usually develops in morbidly obese individual. The underlying pathophysiology is a lymphatic flow disturbance that may be due to the massive amount of adipose tissue [1]. Lymphatic disruption due to previous surgery, trauma, and hypothyroidism also serve as possible causes [2]. The overlying skin usually shows induration and “peau d'orange” appearance consistent with chronic lymphedema and the dermal layer also exhibits stasis dermatitis morphology. It may clinically mimic well-differentiated liposarcoma or other lipogenic tumors which may lead to error in diagnosis.

MLL most commonly occurs in the medial aspect of proximal thigh. Abdominal wall, scrotum, and upper extremity, although rare, are the other possible areas of occurrence. We present an abdominal MLL clinically mimicking well-differentiated liposarcoma from an obese patient which can be excluded by histological and immunohistochemical characteristics. The literature search and differential diagnosis from other mimickers will also be discussed.

## 2. Materials and Methods

### 2.1. Clinical Data

A fifty-six-year-old obese male patient (155 kg), with previous medical history of type II diabetes, hypertension, and dilative cardiomyopathy, presented to our surgery department with a 26 × 22 × 9.5 cm sized, polypoid subcutaneous mass below his umbilicus over 4 years. It was soft in consistency and painless. The overlying skin was thicker with “peau d'orange-” like appearance. Neither previous operation nor history of hypothyroidism was claimed. No sign of hypothyroidism was identified during the clinical examination. Due to the large size of the tumor, the patient underwent surgical excision under the impression of sarcoma.

### 2.2. Immunohistochemistry

After sectioning of 4 *µ*m slides from the formalin-fixed, paraffin-embedded blocks, deparaffinization in xylene and rehydration in a series of decreasing concentration of ethanol were done. Antigen retrieval using either the Bond Epitope Retrieval Solution 1 (pH 6) or the Bond Epitope Retrieval Solution 2 (pH 9) (Leica Microsystems, Wetzlar, Germany) at 99-100°C for 20–30 min was performed. The slides were treated with S-100 (1 : 300, clone S-100, DAKO, Carpinteria, California, USA, incubation time: 30 min in room temperature), vimentin (prediluted, clone V91, DAKO, incubation time: 30 min in room temperature), CD31 (1 : 30, clone JC70A, DAKO, incubation time: 30 min in room temperature), MDM2 (1 : 200, clone IB10, Novocastra, Wetzlar, Germany, incubation time: 45 min in room temperature), and p16 (1 : 10, clone G175-405, DAKO, incubation time: 30 min in room temperature), separately. Immunostaining was performed on Leica BOND-MAX autostainer (Leica Microsystems) and we used peroxidase/DAB Bond Polymer Refine Detection System (Leica Microsystems) for visualization. Cytoplasmic staining for vimentin and CD31 and nuclear staining for MDM2 are regarded as positive stain whereas, for S-100 and p16, both nuclear and cytoplasmic stains are required.

## 3. Results

### 3.1. Gross Findings

**Figure 1:**
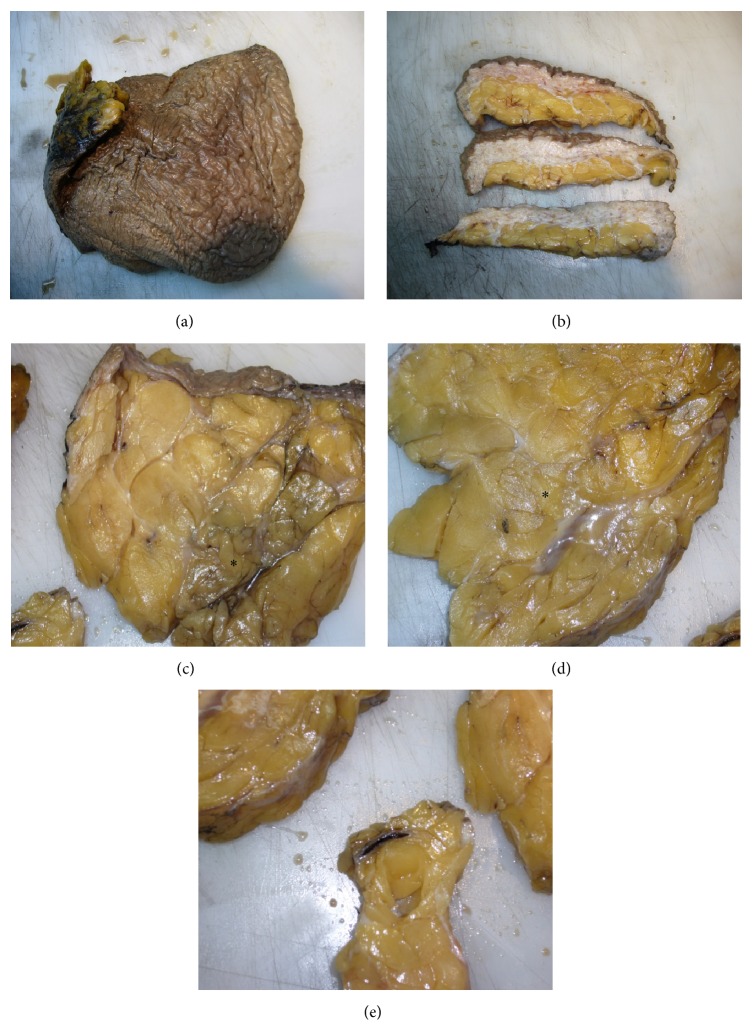
Macroscopic characteristics of MLL. The skin is indurated and hyperpigmented with classical “peau d'orange” appearance (a). The cut surface revealed the thickened skin (b). The subcutaneous tissue cut surface showed serous fluid (indicated by “*∗*”) and also lobulated proliferation of fatty tissue (c) partially surround by thickened fibrous bands ((d), indicated by “*∗*”). Focal dilated, thrombotised vessels were also noted (e).

During pathological examination, a polypoid skin-covering subcutaneous lesion was received measured 26 × 22 × 9.5 cm in size. The skin was indurated and hyperpigmented with classical “peau d'orange” appearance (Figure 1(a)). On cut surface revealed the thickened skin (Figure 1(b)). The subcutaneous tissue cut surface revealed clear, serous-like fluid and also massive amount of fat tissue partially surrounded and traversed by thickened fibrous bands (Figures 1(c)-1(d)). Focal dilated, thrombotised vessels were also noted (Figure 1(e)).

### 3.2. Histopathological Findings

**Figure 2:**
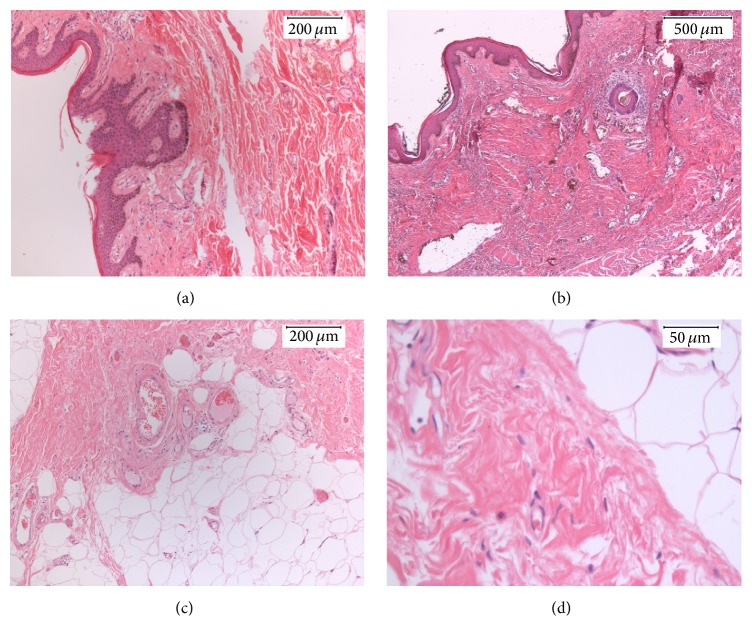
Histomorphology of MLL. The epidermis showed mild acanthosis, elongated rete ridges, basal melanocytic proliferation, and hyperpigmentation ((a), 20x). The dermis revealed reactive lobular vascular proliferation with congestion and mild lymphocytic infiltration similar to stasis dermatitis ((b), 10x). The subcutaneous areas show adipose tissue and reactive vascular proliferation ((c), 20x) and reactive fibroblasts ((d), 40x).

**Figure 3:**
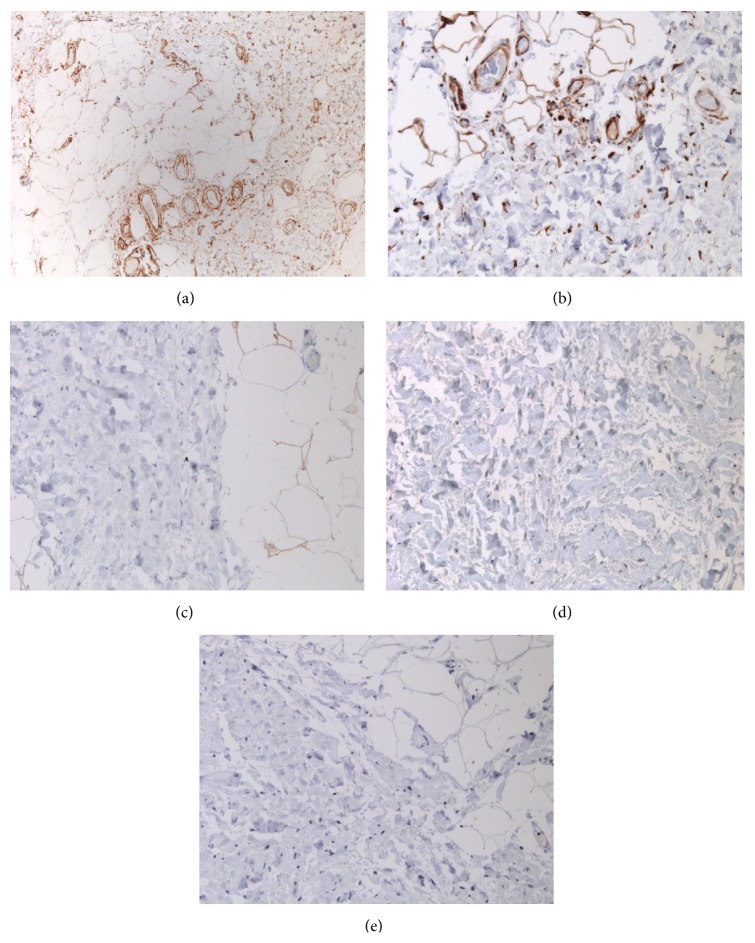
Reactive lobular vascular proliferation, highlighted by CD31 ((a), 10x). The stromal cells are positive for vimentin ((b), 20x) and negative for S-100, MDM2, and p16 ((c)–(e), all 20x).

Microscopically, the epidermis showed mild acanthosis, elongated rete ridges, basal melanocytic proliferation, and hyperpigmentation (Figure 2(a)). The dermis revealed reactive lobular congested vascular proliferation (Figure 2(b)) with mild lymphocyte filtration and fibrosis similar to stasis dermatitis. Subcutaneous tissue showed lobulated adipose tissue proliferation traversed by expanded, edematous subcutaneous septa. Scattered reactive fibroblasts are seen; however, the atypical, hyperchromatic stromal cells and lipoblasts, typically seen in well-differentiated liposarcomas, are not identified. Reactive fibroblast and vascular proliferation surrounded the fatty lobules (Figures 2(c) and 2(d)); the latter was also highlighted by CD31 (Figure 3(a)). Mitosis and necrosis were not seen.

Immunohistochemically, the reactive fibroblast cells were stained for vimentin (Figure 3(b)) and they were negative for S-100, MDM2, and p16 (Figures 3(c)–3(e)).

### 3.3. Clinical Follow-Up Data

The patient underwent follow-up control for 8 months since the operation. No recurrence of the lesion was found and plastic surgery was advised.

## 4. Discussion

MLL is a rare clinical entity that has relatively few literature published [1, 3, 4]. It is usually associated with lymphedema from variable causes which may lead to lymphatic obstruction and lymphedema. It usually occurs in morbidly obese patient by the weight of large amount of fat which may create an ischemic microenvironment and stimulate a wound-like process that recruits growth factors to the area [2]. The association between MLL and hypothyroidism had been reported as well [2]. Women are more commonly affected than men. The average size of the lesion is 28.5 cm ranging from 19.5 to 61.5 cm [1]. MLL are frequently present for a considerable time before resection and may only reach clinical attention when they interfere with daily activities or are secondarily inflamed. The radiological study usually revealed expanded subcutaneous tissue with soft tissue bands but without a discrete mass lesion which may indicate its adipose tissue nature. The most common location is around the medial aspect of thigh. Recently several reports have documented that MLL can also occur in other areas such as vulva, mons pubis, scrotum, and upper extremity [5–9]. Abdominal localization is relatively rare and clinically is usually underdiagnosed as malignant tumor, particularly well differentiated liposarcoma (WDLPS), based on clinical and radiological impressions [10].

Indeed, clinical pictures and histologically expanded interlobular septae and massive amount of adipose tissue may mimic WDLPS; nevertheless, lymphedema is a superficial process that involves mainly skin and dermo-/epidermochanges related to chronic lymphedema without rapid growth imply a benign process. WDLPS usually forms a discrete lesion, as opposed to the diffuse changes seen in MLL. In microscopic levels, the presence of widened, edematous fibrous septa together with reactive fibroblasts may mimic WDLP.

In MLL, the adipocytes are evenly sized and lack the characteristic hyperchromatic stromal cells, thick-walled blood vessels containing similar hyperchromatic cells within their walls, and lipoblasts. In challenge cases, immunohistochemical stain for MDM2, CDK4, and p16 [11] or fluorescent in situ hybridization to detect MDM2 gene amplification can confidently separate MLL from WDLPS. Lack of plexiform-type capillaries, myxoid stroma, and lipoblast/round cell can also rule out myxoid liposarcoma. On demand, fluorescent in situ hybridization to detect t(12;16) (*FUS*;*CHOP*) translocation can be used as a confirmation test.

Desmoid-type fibromatosis may also be one of differential diagnoses due to the reactive fibroblasts and fibroedematous stroma. However, similar to WDLPS, desmoid-type fibromatosis usually presents as a discrete fibrous lesion within the soft tissues with long, sweeping fascicles of bland (myo)fibroblasts together with thin-walled, dilated vasculature and perivascular oedema and peripheral chronic inflammatory cell infiltration which cannot be found in MLL. Negative nuclear stain of *β*-catenin of fibroblast in MLL can also serve as a great diagnostic clue.

Low grade myxofibrosarcoma occurs usually in elderly patient's extremities. It can be differentiated from MLL by its relatively localized nature, atypical hyperchromatic spindle cells, myxoid stroma, and well-developed arcing vasculature.

Since the underlying cause of MLL is lymphatic obstruction possibly due to morbid obesity, persistence or even recurrence of these lesions is expected after the surgical resection although recurrence with an aggressive manner has not been documented [1]. Recently, cases of squamous cell carcinoma and cutaneous angiosarcoma arising from long-standing MLL have been reported [12, 13] probably through similar pathomechanism as Stewart–Treves syndrome. Hence long term follow-up is still recommended.

In conclusion, we present a case of MLL arising from abdominal wall from a morbidly obese patient which is a rare pseudosarcomatous lesion and diagnosis can be challenging for pathologists. Awareness of this rare entity and detailed clinical and radiological information and correlation are required. Differential diagnosis of WDLPS, the most common mimicker, can be ruled out by immunohistochemical stains and fluorescent in situ hybridization. Although rare, secondary sarcomatous transformation had been documented; frequent follow-up is recommended.

## Abbreviations

ALT: Atypical lipomatous tumor
IHC: Immunohistochemistry
MLL: Massive localized lymphoedema
WDLPS: Well differentiated liposarcoma.

## References

[1] Manduch M., Oliveira A. M., Nascimento A. G., Folpe A. L. (2009). Massive localised lymphoedema: a clinicopathological study of 22 cases and review of the literature. *Journal of Clinical Pathology*.

[2] Wu D., Gibbs J., Corral D., Intengan M., Brooks J. J. (2000). Massive localized lymphedema: additional locations and association with hypothyroidism. *Human Pathology*.

[3] Evans R. J., Scilley C. (2011). Massive localized lymphedema: a case series and literature review. *Canadian Journal of Plastic Surgery*.

[4] Farshid G., Weiss S. W. (1998). Massive localized lymphedema in the morbidly obese: a histologically distinct reactive lesion simulating liposarcoma. *The American Journal of Surgical Pathology*.

[5] Bognár G., Novák A., István G. (2014). Massive localized lymphoedema (MLL) in the mons pubis. *Magyar Sebészet*.

[6] Fife C. (2014). Massive localized lymphedema, a disease unique to the morbidly obese: a case study. *Ostomy Wound Management*.

[7] Heller D. S., Fitzhugh V. A., Cracchiolo B., Barrett T., Suidan R. S. (2014). Massive localized lymphedema of the vulva: report of a case and review of the literature. *Journal of Lower Genital Tract Disease*.

[8] Jones M., Marshall D., Mason D. (2011). Massive localized lymphoedema. *ANZ Journal of Surgery*.

[9] Lee S., Han J. S., Ross H. M., Epstein J. I. (2013). Massive localized lymphedema of the male external genitalia: a clinicopathologic study of 6 cases. *Human Pathology*.

[10] El-Sharkawy M. S., Al-Rikabi A. C., Alarfaj N., Al Mugaren F. M. (2014). Localized massive lymphedema masquerading as an anterior abdominal mass mimicking a liposarcoma. *The American Journal of the Medical Sciences*.

[11] Thway K., Flora R., Shah C., Olmos D., Fisher C. (2012). Diagnostic utility of p16, CDK4, and MDM2 as an immunohistochemical panel in distinguishing well-differentiated and dedifferentiated liposarcomas from other adipocytic tumors. *The American Journal of Surgical Pathology*.

[12] Shon W., Ida C. M., Boland-Froemming J. M., Rose P. S., Folpe A. (2011). Cutaneous angiosarcoma arising in massive localized lymphedema of the morbidly obese: a report of five cases and review of the literature. *Journal of Cutaneous Pathology*.

[13] Su T., Lee H., Gao H., Nieh S., Lin C. (2015). Squamous cell carcinoma arising from massive localized lymphedema of scrotum mimicking scrotal smooth muscle hamartoma of darto: a case report. *The American Journal of Dermatopathology*.

